# Identification and Expression Patterns of Critical Genes Related to Coat Color in Cashmere Goats

**DOI:** 10.3390/genes16020222

**Published:** 2025-02-14

**Authors:** Dubala Wu, Jing Fan, Yue Pang, Binhong Wen, Wei Li, Guanghao Yang, Huiyu Cheng, Jiahui Shi, Ting Wang, Sile Hu, Chun Li, Bin Liu, Jun Yin, Jianghong Wu

**Affiliations:** 1 College of Animal Science and Technology, Inner Mongolia Minzu University, Tongliao 028000, China; yinghua19870901@163.com (D.W.); 13171423726@163.com (J.F.); pangyue515@163.com (Y.P.);; 2 College of Life Science, Inner Mongolia Minzu University, Tongliao 028000, China; 3 Institute of Animal Husbandry, Inner Mongolia Academy of Agricultural & Animal Husbandry Sciences, Hohhot 010031, China; 4 College of Animal Science, Inner Mongolia Agricultural University, Hohhot 010018, China

**Keywords:** cashmere goat, coat color, critical genes, expression patterns, melanin synthesis

## Abstract

**Background/Objectives:** Research on cashmere goat coat color is crucial for optimizing cashmere goat breeds and increasing their economic value. To identify key genes associated with the formation of cashmere goat coat color and to provide molecular markers for breeding purposes, three healthy, 3-year-old does with similar weights and distinct coat colors—white, black, and light brown—were selected. **Methods:** Skin samples were collected for transcriptome sequencing, and bioinformatics methods were applied to screen for differentially expressed genes (DEGs) in the skin of cashmere goats with varying coat colors. Real-time fluorescence quantitative PCR (qRT-PCR) and immunofluorescence were subsequently conducted to examine the expression patterns of these DEGs. **Results:** The results showed that a total of 1153 DEGs were identified across the three groups of cashmere goats. According to GO and KEGG analyses, these DEGs were involved in key biological processes and structures, such as the melanin biosynthetic process (GO:0042438), melanosome membrane (GO:0033162), and melanin biosynthesis from tyrosine (GO:0006583). Employing Cytoscape, a gene interaction network was plotted, highlighting a compact network of DEGs associated with coat color formation. Critical genes identified included *TYRP1*, *TYR*, *DCT*, *ASIP*, *PMEL*, *LOC102180584*, *MLANA*, *TSPAN10*, *TRPM1*, *CLDN16*, *AHCY*, *LOC106503350*, and *LOC102175263*. qRT-PCR and fluorescence immunohistochemistry further determined that TYRP1, TYR, DCT, and PMEL expression levels were high in black goats (BGs), while ASIP and AHCY expression levels were high in white goats (WGs). The expression levels of these six genes in light brown goats (RGs) were intermediate between those in BGs and WGs. **Conclusions:** *TYRP1*, *TYR*, *DCT*, and *PMEL* were believed to play pivotal roles in the formation of black coat color, while *ASIP* and *AHCY* regulated the formation of white coat color in cashmere goats.

## 1. Introduction

Cashmere goats are a specialized breed primarily raised for producing high-quality cashmere fiber. Of them, the Inner Mongolia breed is globally renowned for its exceptional cashmere production and the superior quality of its fine fibers. While most cashmere goats are predominantly white, black and light brown populations also exist. These goats are notable for their robust build, excellent cashmere quality, high meat yield, and outstanding adaptability to arid climates and the harsh ecological conditions of barren pastures and hilly grasslands.

Coat color is a prominent phenotype in animals, playing a crucial role in genetic and evolutionary processes. This phenotype is closely associated with essential survival functions such as attracting mates, communication, and predator avoidance [1,2]. In livestock species such as cashmere goats, sheep, and alpacas, coat color is also an economically significant trait. Natural coat colors are particularly valuable for producing dye-free fleece products [3,4]. The type and quantity of melanin deposited determine mammalian skin and coat color. Melanin, a pigment produced by melanocytes, is primarily found in hair follicles and the skin’s epidermis [5]. Several signaling pathways and genes regulate melanocyte development, including EDN3/EDNRB, KIT/KITL, and Wnt signaling pathways, which are crucial for the differentiation and development of neural crest cells into melanocytes. Moreover, transcription factors such as MITF, PAX3, and SOX10 play pivotal regulatory roles in melanocyte differentiation and development [6,7]. *EDN3* is essential throughout melanocyte development and partially compensates for *KIT*’s role during early differentiation stages, thereby assisting in melanin production. *EDN3* overexpression reduced white spots in mice with KIT mutations [8]. During melanoblast migration, KIT binds to its ligand KITL, triggering Ser73 phosphorylation on MITF-M via mitogen-activated protein kinases (MAPKs), which enhances MITF-M activity. The transcriptional coactivator CBP/p300 further interacts with MITF-M, amplifying its function [9]. Mutations in *KIT* that reduce its normal expression disrupt melanocyte migration, resulting in diluted coat color and potentially causing white coat color or white spots caused by a lack of melanocytes in hair follicles [9,10]. The Wnt signaling pathway promotes melanoblast proliferation and differentiation and enhances melanin synthesis by accumulating β-catenin in melanocytes. β-catenin then translocates to the nucleus, where it interacts with lymphocyte enhancer factor 1 (LEF1) to upregulate *MITF* transcription [11].

Melanosomes are membrane-bound organelles within melanocytes and play a crucial role in melanin synthesis. They originate from the Golgi apparatus, endoplasmic reticulum, and lysosomes. The premelanosome (PMEL) protein, specific to melanocytes, regulates melanosome morphogenesis by forming physiological amyloid fibrils. The PMEL protein is synthesized in the endoplasmic reticulum and undergoes processing and modification in the Golgi apparatus and Golgi network structure before entering melanosomes. Mutations or inactivation of the *PMEL* gene alter melanosome morphology, leading to coat color dilution [12].

Melanocytes produce two primary forms of melanin: eumelanin, which gives skin and hair black or brown tones, and pheomelanin, responsible for red and yellow hues [13]. These pigments are synthesized through distinct pathways: In melanosomes, tyrosine is converted to DOPA by tyrosinase (TYR). DOPA is then oxidized to form DOPA quinone (DQ) and subsequently converted into dopachrome. The enzyme DCT transforms dopachrome into 5,6-dihydroxyindole carboxylic acid (DHICA) and 5,6-dihydroxyindole (DHI), while TYRP1 oxidizes DHICA and DHI to produce eumelanin [14]. In the presence of cysteine, ASIP and α-MSH competitively interact with MC1R, inhibiting TYR expression and blocking eumelanin production. This favors pheomelanin synthesis through loop closure and decarboxylation reactions [15]. Eumelanin and pheomelanin production are also affected by environmental pH within melanosomes. Lower pH levels favor pheomelanin production, whereas higher pH levels promote eumelanin production [16]. Once synthesized, mature melanosomes transport melanin to surrounding keratinocytes via molecular motors, where the pigment performs its function [16,17].

The coat color of cashmere goats directly impacts their market value. While white cashmere is the most sought-after, naturally colored populations, such as brown and cyan, have distinct niche markets. These naturally pigmented cashmere fibers are highly valued for producing specialized products [18]. Therefore, studying the genetic basis of coat color in cashmere goats is essential for understanding their biological diversity and enabling molecular-assisted breeding strategies. Based on prior research and findings from other species, we hypothesized that variations in coat color among cashmere goats result from differences in melanin synthesis in the skin, which is regulated by multiple genes. To test this hypothesis, skin samples were collected from white, black, and light brown cashmere goats for transcriptome sequencing. This study identifies critical genes involved in coat color formation and explores their expression patterns, providing a foundation for the genetic improvement of cashmere goats with diverse coat colors.

## 2. Materials and Methods

### 2.1. Skin Sample Preparation for RNA Sequencing and Validation Experiments

**Figure 1:**
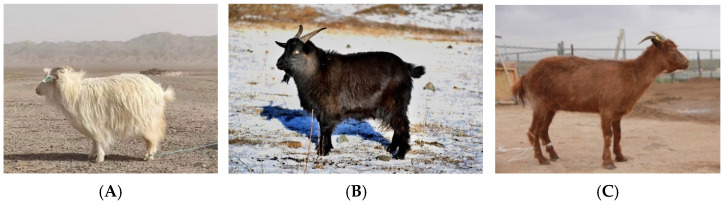
Cashmere goats with different coat colors. (**A**) White cashmere goat, (**B**) black cashmere goat, and (**C**) light brown cashmere goat.

In this study, healthy 3-year-old does with varying coat colors were sourced from the Tonghe Tai Breeding Farm in Urat Left Banner, Bayannaoer City, Inner Mongolia (Figure 1A–C). Skin samples were collected during the anagen phase of hair follicle growth from three white, three black, and three light brown cashmere goats. Three 1 cm^2^ skin samples were collected from the metacarpal region behind the scapula of each cashmere goat. Two of the samples were immediately frozen in liquid nitrogen and stored at −80 °C for RNA extraction for transcriptome sequencing and quantitative real-time PCR (qRT-PCR). The third sample was fixed in freshly prepared 4% paraformaldehyde solution for 24 h, followed by dehydration through a graded alcohol series. Samples from white goats were labeled as W (W_1, W_2, W_3), black goats as B (B_1, B_2, B_3), and light brown goats as R (R_1, R_2, R_3).

### 2.2. RNA Extraction, Library Construction, and RNA Sequencing

Total RNA was extracted from each skin sample by using RNAisoPlus (TAKARA, Kusatsu City, Japan) following the manufacturer’s protocol. RNA concentration and integrity were measured using a NanoDrop 2000c spectrophotometer (Thermo, Waltham, MA, USA). After verifying quality, 3 µg of total RNA from each sample was used for library construction. Due to low RNA quality in one sample from a light brown goat (R_3), only 8 libraries were generated and sequenced using the Illumina HiSeq 2000 platform at CapitalBio Technology Co., Ltd. (Beijing, China).

### 2.3. Quality Control and Alignment of Reads to the Reference Genome

Raw sequencing reads were filtered to remove low-quality sequences, including adapter sequences and regions containing poly-Ns. The clean reads were aligned to the goat reference genome (ARS 1.2) using TopHat software, and the effectively mapped sequencing reads were quantified. Based on the alignment information, HTSeq v0.6.1 was used to calculate the read counts for each transcript, and gene expression levels were quantified using Fragments Per Kilobase of transcript per Million mapped reads (FPKM) values [19]. Genes with FPKM values of >0.01 were considered significantly expressed [20].

### 2.4. Identification and Functional Enrichment Analysis of Differentially Expressed Genes

Differentially expressed genes (DEGs) were identified using the negative binomial distribution model in DESeq v2.0 [21]. Gene expression levels were compared among the three groups. The likelihood ratio test was conducted for hypothesis testing in the following comparisons: white vs. black cashmere goats (WGs vs. BGs), white vs. light brown cashmere goats (WGs vs. RGs), and black vs. light brown cashmere goats (BGs vs. RGs). Significant DEGs were defined as those with a fold change of ≥2.0 and a false discovery rate (FDR) of <0.05.

A hierarchical clustering heatmap was generated using complete linkage and Euclidean distance metrics. Gene Ontology (GO) enrichment analysis was conducted in RStudio, mapping DEGs to functional terms. Hypergeometric tests were used to calculate *p*-values, which were adjusted using the Benjamini–Hochberg procedure. Significantly enriched GO terms were identified based on these corrected *p*-values. Furthermore, pathway enrichment analysis of DEGs was conducted using the Kyoto Encyclopedia of Genes and Genomes (KEGG) database [22], with a significance threshold of *p* < 0.05.

### 2.5. Construction of DEG Interaction Network

To identify key genes involved in melanin synthesis, Cytoscape was employed to construct DEG interaction networks. Correlation coefficients between DEGs were calculated in RStudio. DEGs with correlation coefficients of >0.9 or <−0.9 and *p* < 0.05 were selected for inclusion in the interaction networks.

### 2.6. Validation of Coat Color-Related Key Genes Through qRT-PCR

RNA was extracted using the method described in Section 2.2, and RNA quality was confirmed through 1.0% agarose gel electrophoresis, with images captured using a gel imager. Specific primers for six genes in cashmere goats were designed using Primer Premier 5.0, based on the mRNA coding region sequences of goat genes. All of the primers were handed over to Sangon Biotech (Shanghai) Co., Ltd. (Shanghai, China), and the sequence of the primers is shown in Table 1. High-quality RNA samples were reverse-transcribed into cDNA by using the PrimeScript™ RT Reagent Kit with gDNA Eraser (Perfect Real Time), RR047A (TAKARA, Kusatsu City, Japan). qRT-PCR was conducted using the TB Green Fast qPCR Mix (RR430S, TAKARA, Kusatsu City, Japan). Gene expression levels were calculated using the 2^−ΔΔCt^ method [23].

### 2.7. Identification of Coat Color-Related Key Genes via Skin Tissue Immunofluorescence

Skin tissue samples fixed in 4% paraformaldehyde were dehydrated, embedded in paraffin wax, and cut into 5 µm thick sections. The sections were dewaxed, rehydrated through a graded alcohol series, and subjected to antigen retrieval using sodium citrate. Tissue sections were treated with 0.5% Triton X-100 (Beyotime, Shanghai, China) for 30 min at room temperature and blocked with 10% goat serum (Sangon, Shanghai, China) for 1 h. Primary rabbit-derived primary antibodies (Bioss, Beijing, China) were applied overnight at 4 °C. After the sections were washed three times with PBS, they were incubated with a Coralite488-conjugated secondary antibody (Proteintech, Chicago, IL, USA) for 2 h. Following an additional three washes with PBS, nuclei were stained with DAPI (Beyotime, Shanghai, China), and the sections were mounted with an anti-fade mounting medium and stored in darkness to prevent fluorescence quenching. Images were captured using a fluorescence microscope (Nikon DS-Fi3, Tokyo, Japan). DAPI signals were visualized under ultraviolet light (exposure time: 70–100 ms), while labeled proteins were observed under green excitation light (exposure time: 500–900 ms).

## 3. Results

### 3.1. Summary of the RNA-Seq Data

Transcriptome sequencing of skin samples from three groups of cashmere goats yielded raw read counts ranging from 46,022,200 to 49,144,452. After reads with two or more “N” bases and those with low-quality values were filtered out, clean read counts ranged from 42,903,008 to 45,992,560, representing a clean read proportion of 92.41–94.23%. Alignment with the goat reference genome exhibited a total alignment rate exceeding 96% across all coat color groups. More than 94% of the sequences mapped uniquely to the genome, while approximately 4% aligned to multiple genomic locations (Supplemental Table S1).

### 3.2. Screening of DEGs Between Cashmere Goats with Different Coat Colors

Of the 57,565 goat genes detected, 1153 were identified as DEGs. Between WGs and BGs, 442 DEGs were identified, including 255 upregulated and 187 downregulated genes. In the comparison between WGs and RGs, 372 DEGs were detected, comprising 220 upregulated and 152 downregulated genes. Additionally, 339 significant DEGs were observed between BGs and RGs, of which 170 and 169 genes were upregulated and downregulated, respectively (Table 2).

**Figure 2:**
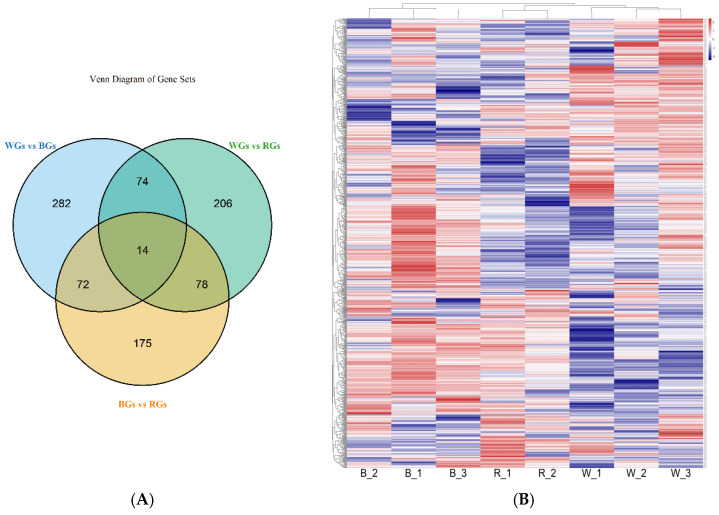
DEGs in three groups of cashmere goats. (**A**) Venn diagram of DEGs and (**B**) DEG cluster-ing heatmap.

Figure 2A illustrates a Venn diagram depicting the shared and unique DEGs across the three coat color groups. The heatmap of gene expression patterns highlights distinct differences in gene expression among WGs, RGs, and BGs. The expression trends in the DEGs were strikingly different between WGs and BGs, whereas those in BGs and RGs were more similar (Figure 2B).

### 3.3. Functional Classification of DEGs

**Figure 3:**
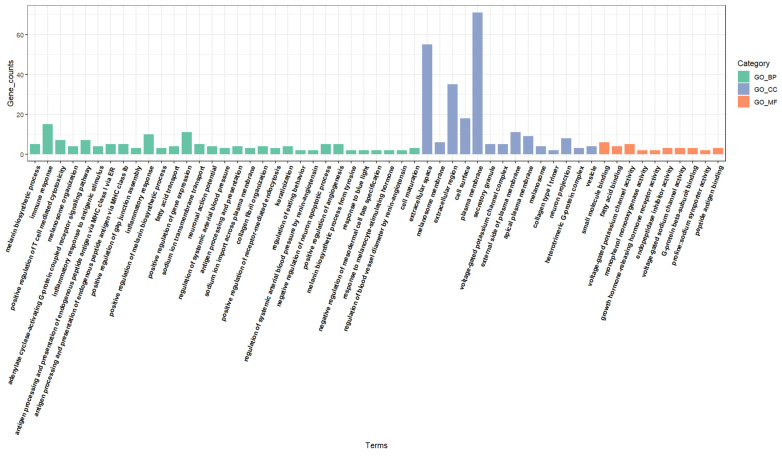
GO function analysis of DEGs.

GO annotation analysis of the DEGs across the three groups identified several significant GO terms (*p* ≤ 0.05) categorized into 31 biological processes (BPs), 14 cellular components (CCs), and 10 molecular functions (MFs) (Figure 3). In the BP functional classification, the annotated genes were predominantly involved in processes such as melanin the biosynthetic process (GO:0042438), immune response (GO:0006955), and positive regulation of T cell-mediated cytotoxicity (GO:0001916). Among these, melanin synthesis-related specific pathways included melanosome organization (GO:0032438), positive regulation of the melanin biosynthetic process (GO:0048023), and the melanin biosynthetic process from tyrosine (GO:0006583). In the CC functional classification, genes were predominantly associated with the extracellular space (GO:0005615), melanosome membrane (GO:0033162), and extracellular region (GO:0005576). Notably, the terms melanosome membrane (GO:0033162) and melanosome (GO:0042470) were particularly relevant to melanin synthesis. The MF classification highlighted activities such as small molecule binding (GO:0036094), fatty acid binding (GO:0005504), and voltage-gated potassium channel activity (GO:0005249).

The KEGG pathway analysis unveiled significant enrichment in 20 signaling pathways (Supplemental Figure S1A–C). DEGs were enriched in pathways related to chemokine signaling, melanogenesis, leishmaniasis, graft-versus-host disease, allograft rejection, tyrosine metabolism, the renin–angiotensin system, and retinol metabolism.

### 3.4. Identification of Critical Genes Associated with Different Coat Colors

**Figure 4:**
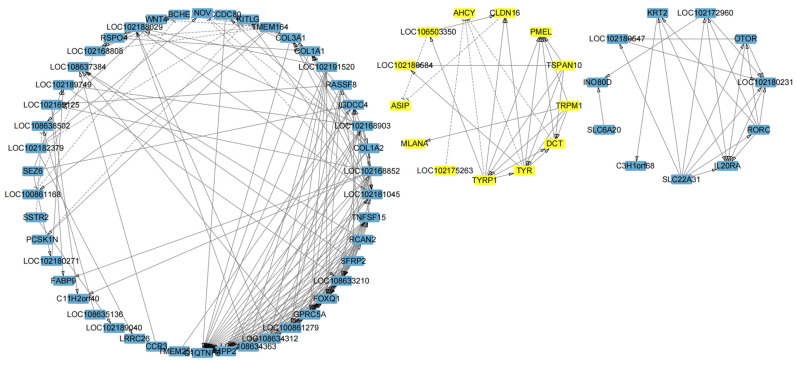
Interaction network of DEGs. Genes related to melanin synthesis-related genes were plot-ted with yellow boxes. The network shows various interactions among these DEGs: → denotes a promoting effect between genes, while --- indicates an inhibitory effect.

By constructing a gene interaction network diagram using Cytoscape, we identified that the DEGs formed a primary network and two smaller sub-networks (Figure 4). Notably, one of the smaller sub-networks was associated with melanin synthesis which constituted and included genes such as *TYRP1*, *TYR*, *DCT*, *ASIP*, *PMEL*, *LOC102180584*, *MLANA*, *TSPAN10*, *TRPM1*, *CLDN16*, *AHCY*, *LOC106503350*, and *LOC102175263* (Table 3). Gene interaction analysis of this sub-network unveiled inhibitory relationships among *TYRP1*, *TYR*, *DCT*, *LOC102175263*, and *AHCY*. Similarly, *CLDN16*, *LOC106503350*, and *ASIP* also exhibited inhibitory interactions. By contrast, *TYRP1*, *TYR*, *DCT*, *TSPAN10*, and *TRPM1* promoted *PMEL* expression. This visualization underscores the complex relationships and interactions between genes involved in melanin synthesis and highlights their functional roles in influencing coat color.

**Figure 5:**
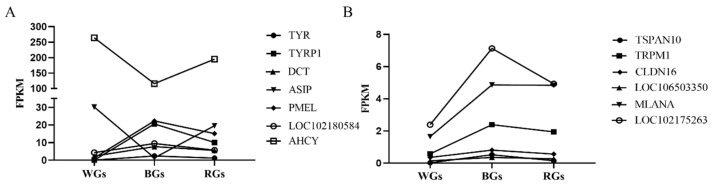
Gene expression trends in the critical genes of cashmere goats with different coat colors. (**A**) shows the FPKM values of *TYR*, *TYRP1*, *DCT*, *ASIP*, *PMEL*, *LOC102180584*, and *AHCY*; (**B**) shows the FPKM values of *TSPAN10*, *TRPM1*, *CLDN16*, *LOC106503350*, *MLANA*, and *LOC102175263*.

Expression trend maps for 13 coat color-related critical genes in cashmere goats were generated using FPKM values (Figure 5A,B). Upon analyzing these maps, we found that the FPKM values of *TYRP1*, *TYR*, *DCT*, *PMEL*, *LOC102180584*, *TSPAN10*, *TRPM1*, *CLDN16*, *LOC106503350*, and *LOC102175263* were higher in BGs than in WGs and RGs. The FPKM values of *DCT*, *TYRP1*, and *PMEL* were significantly higher in BGs than in WGs. By contrast, *ASIP* and *AHCY* expression levels were significantly elevated in WGs compared with BGs and RGs. These findings suggest that genes such as *TYRP1*, *TYR*, *DCT*, *PMEL*, *LOC102180584*, *TSPAN10*, *TRPM1*, *CLDN16*, *LOC106503350*, *MLANA*, and *LOC102175263* are crucial for dark coat color development in cashmere goats, whereas *ASIP* and *AHCY* are key regulators of the formation of white coat colors.

### 3.5. Validation of mRNA Expression Levels of Critical Genes

**Figure 6:**
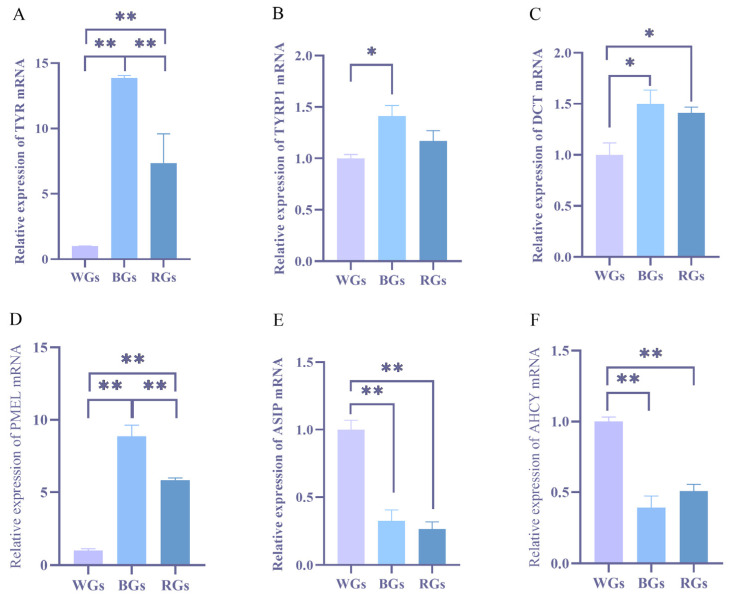
(**A**–**F**) represent the mRNA relative expression of *TYR*, *TYRP1*, *DCT*, *PMEL*, *ASIP*, and *AHCY* (*n* = 3). Data are presented as least squares means ± SEM. * denotes a significant difference at *p* ≤ 0.05; ** indicates an extremely significant difference at *p* ≤ 0.01.

*TYRP1*, *TYR*, *DCT*, *ASIP*, *PMEL*, and *AHCY* mRNA expression levels in the skin of cashmere goats with different coat colors were analyzed using qRT-PCR (Figure 6A–F). *TYR*, *PMEL*, and *DCT* expression levels were notably elevated in BGs and RGs compared with WGs (*p* < 0.01 and *p* < 0.05). The *TYRP1* expression level was higher in BGs than in WGs (*p* < 0.05), whereas no significant difference was observed between the other groups (*p* > 0.05). Conversely, *ASIP* and *AHCY* exhibited higher expression levels in WGs than in BGs and RGs (*p* < 0.01). The qRT-PCR results mostly agreed with the transcriptome sequencing results.

### 3.6. Immunohistochemical Analysis of Critical Genes

**Figure 7:**
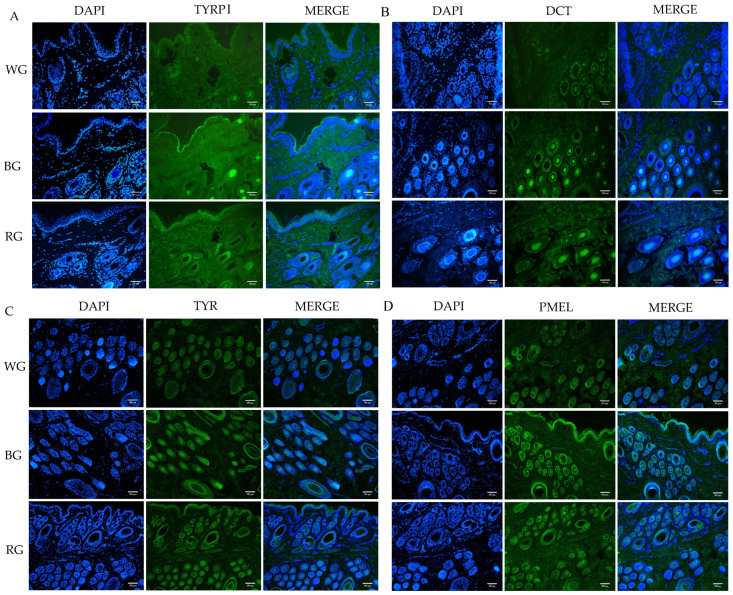

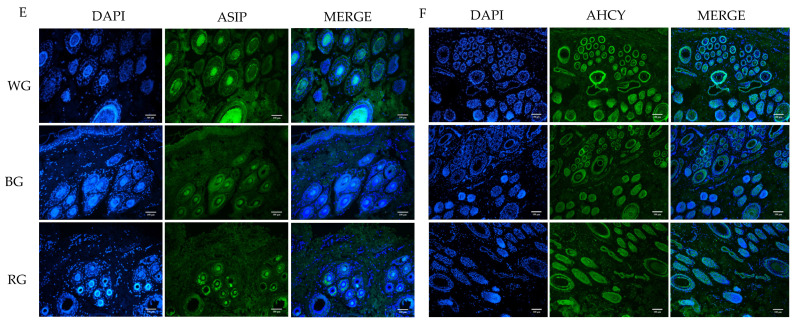
Immunofluorescence for TYRP1 (**A**), DCT (**B**), TYR (**C**), PMEL (**D**), ASIP (**E**), and AHCY (**F**) in skin and hair follicle sections of cashmere goats having different coat colors. Fluorescence signal DAPI (blue) and TYRP1, DCT, TYR, PMEL, ASIP, and AHCY (green). Scale bar = 100 μm.

The protein expression and distribution of the critical melanin-related genes *TYRP1*, *DCT*, *TYR*, *PMEL*, *ASIP,* and *AHCY* in the skin and hair follicles of BGs, RGs, and WGs were analyzed. Immunofluorescence analyses unveiled that TYRP1, DCT, TYR, and PMEL were present in the hair follicles of the three groups of cashmere goats, but the fluorescence signal was strongest in BGs. Of them, TYRP1 and DCT were expressed in the epidermis, hair follicle cortex, and outer root sheath, with stronger signals observed in the cortex than in the outer root sheath (Figure 7A,B). TYR and PMEL were also expressed in the epidermis, hair follicle cortex, inner root sheath, and outer root sheath (Figure 7C,D). By contrast, ASIP and AHCY expression signals were strongest in WGs. ASIP and AHCY were expressed in the hair follicle cortex and outer root sheath (Figure 7E,F).

## 4. Discussion

Melanin plays a crucial role in determining animal coat color, as it is regulated by a complex network of genes that control melanocyte differentiation, maturation, synthesis, and transport [24]. This study conducted a comparative transcriptome analysis of skin samples from cashmere goats with white, black, and light brown coat colors, identifying 1153 DEGs across the three groups. GO analysis revealed significant enrichment of genes associated with BPs involved in melanin biosynthesis. Using Cytoscape, we identified a compact, independent interaction network comprising 13 genes: *TYRP1*, *TYR*, *DCT*, *ASIP*, *PMEL*, *LOC102180584*, *MLANA*, *TSPAN10*, *TRPM1*, *CLDN16*, *AHCY*, *LOC106503350*, and *LOC102175263*. This network highlights mutual and intricate regulatory relationships among these genes associated with melanin synthesis, with *TYRP1*, *TYR*, *DCT*, *TRPM1*, and *TSPAN10* positively influencing *PMEL* expression. By contrast, inhibitory interactions were observed among *TYRP1*, *TYR*, *DCT*, *LOC102175263*, and *AHCY*, as well as among *CLDN16*, *LOC106503350*, and *ASIP*. These findings indicate that *ASIP* and *AHCY* were expressed at low levels in BGs, whereas *TYRP1*, *TYR*, *DCT*, and *PMEL* were expressed at high levels.

In melanosomes, melanin production begins with tyrosinase (TYR) catalyzing the oxidation of tyrosine to DOPA and subsequently to DQ. Further enzymatic reactions yield eumelanin, while cysteine, when present, interacts with DQ to form cysteine DOPA, which undergoes a closure and carboxyl group removal to yield pheomelanin [25,26]. In our study, qRT-PCR analysis revealed significantly higher expression of *TYR*, *DCT*, and *TYRP1* in BGs than in WGs and RGs, which is consistent with findings in sheep with varying coat colors [27]. Immunohistochemical analysis demonstrated the presence of TYRP1 and DCT in the epidermis, outer root sheath of the hair follicle, and hair cortex, with TYR also expressed in the inner root sheath during the anagen phase. Natalia V. et al. observed that TYRP1 and DCT were also found in the outer root sheath and dermal papilla cells of mouse hair follicles during anagen [28]. This expression may be related to increased follicular melanogenesis (FM) during anagen, decreased FM synthesis during catagen, and the absence of FM during telogen [29]. Fluorescence signal intensity data were unveiled and confirmed the strongest signals for TYR, TYRP1, and DCT in BGs, correlating with elevated melanin levels in their skin and hair follicles.

The PMEL protein is specific to pigment cells and vital for forming fibrils within melanocyte organelles, which are essential for pigment cell function. These fibrils serve as scaffolds for melanin polymerization during melanin synthesis [30]. Mutations or the absence of PMEL results in reduced pigment cell viability and lower eumelanin content in hair [12]. For instance, the PMEL p.Leu18del mutation in the Kumamoto sub-breed of Japanese Brown cattle leads to diluted coat color. Individuals having heterozygous genotypes display intermediate coat color with varying dilutions, whereas wild-type cattle have a brown coat, suggesting that PMEL polymorphism can be used as a DNA marker for controlling cattle coat color [31]. In our study, *PMEL* was predominantly enriched in BPs related to melanin biosynthesis and its positive regulation, with the highest expression observed in BGs. Significant differences were observed among the three coat color types. Immunohistochemistry revealed PMEL localization in the epidermis, outer and inner root sheaths of the hair follicle, and hair cortex, which is consistent with findings in Pashmina goats wherein Basharat Bhat et al. witnessed similar PMEL expression patterns in black, white, and brown goats [32].

*ASIP* is pivotal in regulating melanocyte development and melanin production, especially in establishing the white coat phenotype. RNA-seq data from swamp buffalo unveiled that skin *ASIP* levels were 10.3 times greater in white buffaloes than in black buffaloes. The elevated *ASIP* expression was linked to LINE-1 transposon insertion upstream of the *ASIP* gene, which enhanced its expression and subsequently inhibited melanin synthesis, resulting in the white phenotype [33]. Similarly, our study found significantly elevated *ASIP* expression in WGs compared with the other two cashmere goat types. This high expression may result from *ASIP*’s interaction with MC1R to inhibit MITF’s function in WGs, leading to pheomelanin production by melanocytes, which thus results in the white phenotype [34]. Localization analysis revealed ASIP in the hair follicle cortex and outer root sheath membrane, with the strongest fluorescence signals observed in WGs. This may be attributable to the high levels of ASIP regulators, such as Attractin (Atrn) and Mahogunin (Mgrn1), in WGs, which are located downstream of ASIP and upstream of MC1R. In hair follicles, Attractin binds to the amino-terminal domain of the agouti protein, while Mahogunin reduces MC1R activity. Together, Attractin and Mahogunin inhibit the response of hair follicle melanin to ASIP [35].

AHCY encodes the enzyme S-adenosylhomocysteine hydrolase (SAHase). In goats, this gene is located on chromosome 13, downstream of *ASIP*, and is implicated in coat color formation through its role in regulating melanin production [36]. In this study, *AHCY* expression was highest in WGs and lowest in BGs, mirroring its expression trend in *ASIP*. Immunohistochemical analysis revealed that the AHCY protein is localized in the cortex and outer root sheath of hair follicles. These findings suggest that *ASIP* and *AHCY* co-expression inhibit eumelanin synthesis, contributing to the white coat color phenotype. *ASIP* and *AHCY* mutations can lead to protein denaturation and functional loss, resulting in increased melanin synthesis and black hair production. A genome-wide association study conducted by Anahit et al. identified a single-nucleotide polymorphism locus most strongly associated with dark coat colors, specifically black and brown, in Markhoz goats. This locus is located on chromosome 13 within a 465 Kb linkage disequilibrium region containing the *ASIP*, *AHCY*, *RALY*, and *ITCH* genes, underscoring their significance in coat color variation [36]. Additionally, a deletion of 11 bp within exon 2 of *ASIP* in horses (g.2174-2184del) causes a frameshift mutation that results in a recessive allele (a). Homozygosity for this allele (a/a) produces black coat color in horses [37,38].

*MLANA* (*MART1*) is a key gene in melanosome development and plays a critical role in stabilizing PMEL expression in melanosomes. Once MLANA was treated with siRNA, PMEL stability in melanocytes was compromised, impacting its processing and transport [39]. In our study, *MLANA* was enriched in the melanosome, a cellular component, with RNA-seq analysis revealing elevated *MLANA* expression in BGs and RGs compared with WGs. Similarly, Mahanthi Vasu and colleagues reported high *MLANA* expression in Changthangi sheep (black sheep), suggesting that genes like *MLANA*, *TYR*, and *PMEL* are vital for promoting black coat color and providing ultraviolet protection for animals at high altitudes [40]. Furthermore, *MLANA* mutations or knockout decreased melanin synthesis. Mouse studies have reported that the absence of MLANA has no impact on the localization of melanocyte-specific proteins or PMEL17 maturation. However, it does cause morphological abnormalities in capsular melanocytes’ melanosomes. This ultimately reduces the total melanin content in the skin and coat [41].

**Figure 8:**
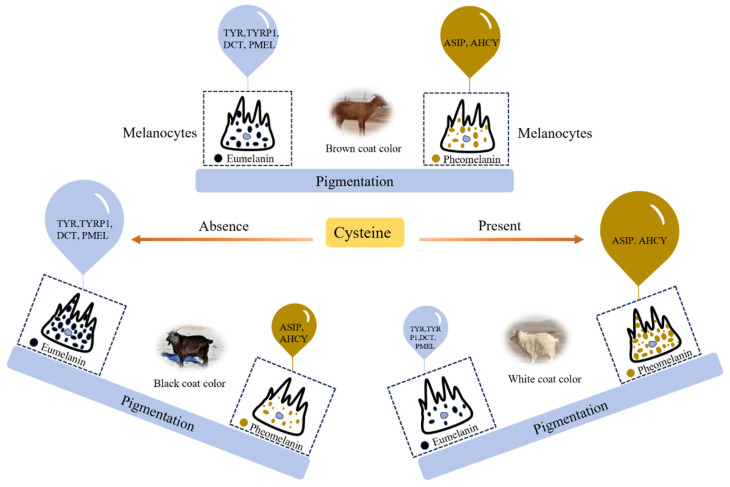
A molecular mechanism model of three coat colors in cashmere goats. The size of the balloon represents gene expression. A larger balloon indicates higher gene expression, leading to increased eumelanin or pheomelanin production by the corresponding melanocytes. Conversely, a smaller balloon represents lower gene expression, resulting in reduced pigment synthesis.

Based on our experimental results, *TYRP1*, *TYR*, *DCT*, and *PMEL* were highly expressed in BGs, with low expression levels in WGs. This differential expression increases eumelanin production by melanocytes, promoting the development of black coat color. Conversely, *ASIP* and *AHCY* exhibited high expression levels in WGs and low levels in BGs, inhibiting pigment synthesis and resulting in white fur coloration. The expression of these six genes in RGs was intermediate between those observed in BGs and WGs (Figure 8).

## 5. Conclusions

In total, 57,565 goat genes were identified in this study, of which 1153 were differentially expressed. Thirteen critical genes associated with coat color formation were identified, including *TYRP1*, *TYR*, *DCT*, *ASIP*, *PMEL*, *LOC102180584*, *MLANA*, *TSPAN10*, *TRPM1*, *CLDN16*, *AHCY*, *LOC106503350*, and *LOC102175263*. qRT-PCR and fluorescence immunohistochemistry further confirmed the high expression levels of *TYRP1*, *TYR*, *DCT*, and *PMEL* in BGs, and those of *ASIP* and *AHCY* in WGs. These results imply that genes such as *TYR, TYRP1, DCT,* and *PMEL* are critical for forming dark coat colors in cashmere goats, whereas *ASIP* and *AHCY* are essential for developing white coat colors. This study offers insights into how gene mutations lead to different coat color phenotypes, potentially aiding in the application of genetic markers for breeding programs. Additionally, the results offer a theoretical foundation for investigating the specific functions of these genes in melanin production and hair follicle development in cashmere goats.

## Figures and Tables

**Table 1:** Sequences of primers for key genes and the reference gene.

Gene Name	Primer Sequences (5′–3′)	Accession Number	Product Length (bp)
*TYRP1*	F: TGGCAATTTCTCAGGACAC R: CTGGACAAAGCGGTTCTT	NM_001285727.1	133
*DCT*	F: TCTGCTGCCAATGATCC R: GGGAAGAAAGGAACCATGT	XM_005687700.3	158
*ASIP*	F: AGTGCCCCACAGTTTTCA R: CAAGGTAGCCAGGAAGAGGT	XM_018057736.1	150
*PMEL*	F: AGGGACCTACTGCCTCAA R: AGCAAGATGCCCACAAAC	XM_018048106.1	180
*AHCY*	F: GGCAAGGTGGCAGTGGTT R: CAGCCTGAAGTGCGTTGAT	XM_018057737.1	121
*TYR*	F: GCGGAAGTTGTAAGTTTGG R: GGGCTGGTGGTATGTTTT	NM_001287562.1	142
*β-actin*	F: GCAAATGCTTCTAGGCGGAC R: TGCTGTCACCTTCACCGTTC	NM_001314342.1	194

**Table 2:** DEGs in skin samples of three groups of cashmere goats.

DEG Sample	Up DEG NO.	Down DEG NO.	Total
WGs vs. BGs	255	187	442
WGs vs. RGs	220	152	372
BGs vs. RGs	170	169	339

**Table 3:** Information about melanin synthesis-related critical genes.

Gene ID	Gene Name	Gene Description
102182281	*TYR*	Tyrosinase
100861390	*TYRP1*	Tyrosinase-related protein 1
102169882	*DCT*	Dopachrome tautomerase
100860915	*ASIP*	Agouti signaling protein
102176102	*PMEL*	Premelanosome protein
102178069	*MLANA*	Melan-A
102181897	*TSPAN10*	Tetraspanin 10
102182733	*TRPM1*	Transient receptor potential cation channel subfamily M member 1
102189431	*CLDN16*	Claudin 16
106503350	*LOC106503350*	Uncharacterized LOC106503350
102175263	*LOC102175263*	Vitamin D3 hydroxylase-associated protein
102180584	*LOC102180584*	BOLA class I histocompatibility antigen

## Data Availability

Transcriptome data are being submitted to the SRA database with the submission number SUB14910368.

## Supplementary Materials

The following supporting information can be downloaded at https://www.mdpi.com/article/10.3390/genes16020222/s1: Supplemental Table S1: Results of transcriptome sequencing quality assessment; Supplemental Figure S1: KEGG enrichment analysis of the three groups.
