# Supplementary material for: Identification and Expression Patterns of Critical Genes Related to Coat Color in Cashmere Goats
Source: Genes (Basel). 2025 Feb 14;16(2):222. doi: 10.3390/genes16020222 (PMC11855694; doi:10.3390/genes16020222)
Supplement: Supplementary file 1 [file genes-16-00222-s001.zip › Supplemental Fig S1.pdf]

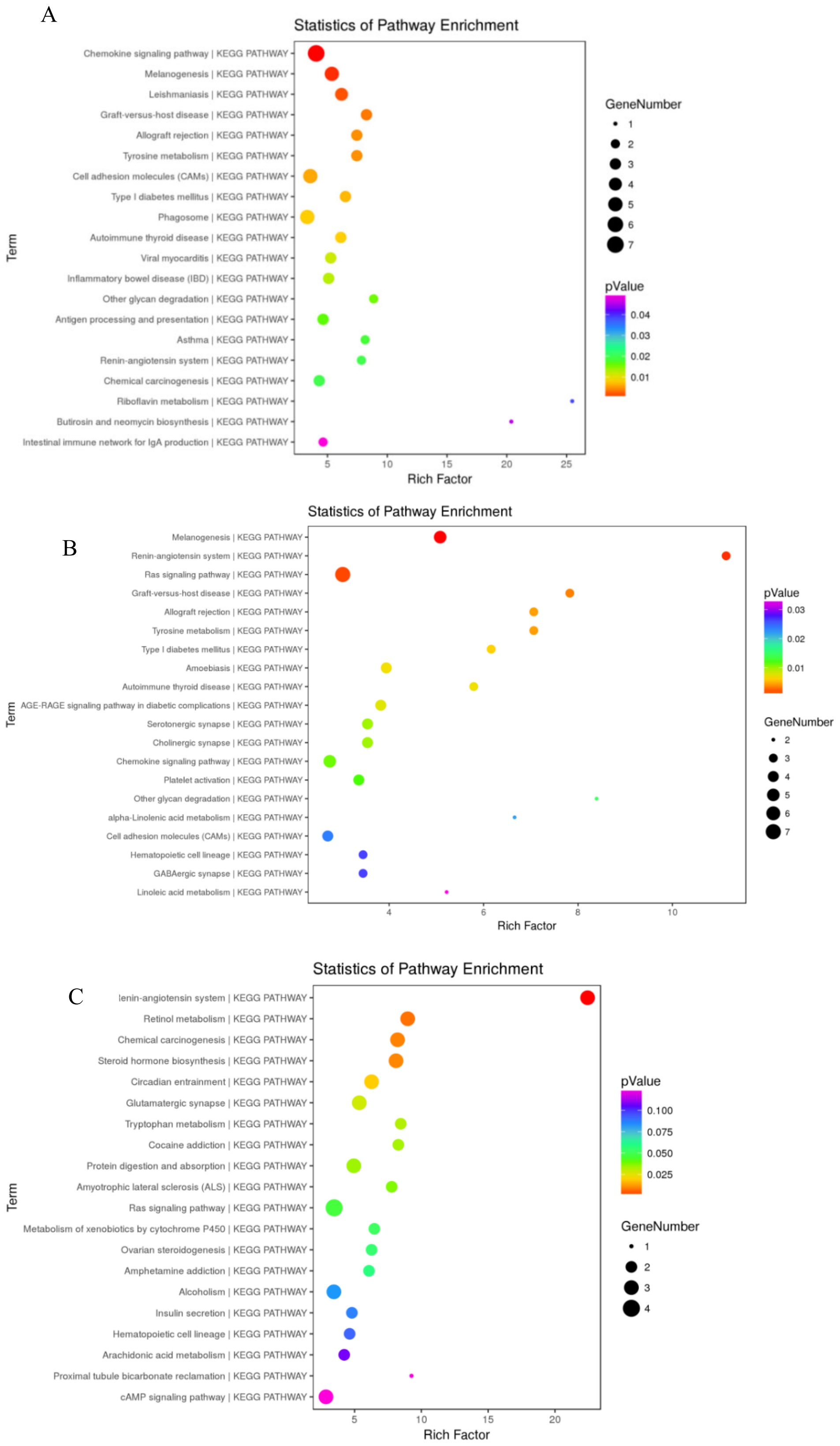

Supplemental Figure S1: A-C: KEGG enrichment analysis of the DEGs in WGs vs. BGs, WGs vs. RGs and BGs vs. RGs.
