# Supplementary material for: Identification and Expression Patterns of Critical Genes Related to Coat Color in Cashmere Goats
Source: Genes (Basel). 2025 Feb 14;16(2):222. doi: 10.3390/genes16020222 (PMC11855694; doi:10.3390/genes16020222)
Supplement: Supplementary file 1 [file genes-16-00222-s001.zip › Supplemental Table S1.pdf]

Supplemental Table S1. Results of transcriptome sequencing quality assessment

Samples	Raw_reads	Clean_reads	Clean_rate(%)	Mapped reads	Uniquely mapped reads	Multiple mapped reads
W_1	47863294	44228594	92.41	42837240(96.85%)	40641604(94.87%)	2195636(5.13%)
W_2	46022200	42903008	93.22	41597316(96.96%)	39409221(94.74%)	2188095(5.26%)
W_3	48408640	45497822	93.99	44023923(96.76%)	41900946(95.18%)	2122977(4.82%)
B_1	48595428	45364436	93.35	43874931(96.72%)	41881759(95.46%)	1993172(4.54%)
B_2	47043782	43939986	93.4	42522294(96.77%)	40540450(95.34%)	1981844(4.66%)
B_3	46681314	43495500	93.18	42141112(96.89%)	40123041(95.21%)	2018071(4.79%)
R_1	49144452	45886258	93.37	44420269(96.81%)	42305941(95.24%)	2114328(4.76%)
R_2	48809168	45992560	94.23	44617956(97.01%)	42450324(95.14%)	2167632(4.86%)

Note: The sample beginning with W is a white cashmere goat, the sample beginning with B is a black cashmere goat, and the sample beginning with R is a light brown cashmere goat.
